# Spontaneous Aryl Diazonium Treatment of Exposed Carbon Fiber Surfaces for Reducing Galvanic Corrosion at Composite-Aluminum Alloy Joints during Cyclic Accelerated Degradation Testing

**DOI:** 10.1021/acsomega.6c00063

**Published:** 2026-05-06

**Authors:** Isuri N. Dammulla, Greg M. Swain

**Affiliations:** Department of Chemistry, 3078 Michigan State University, 578 South Shaw Lane, East Lansing, Michigan 48824, United States

## Abstract

The treatment of exposed carbon surfaces in a carbon fiber reinforced epoxy composite (CFRP) with a spontaneously formed adlayer of 4-nitroazobenzene (NAB) diazonium was applied to inhibit galvanic corrosion on a mechanically joined and conversion-coated aluminum alloy 7075-T6. The adlayer was formed by immersion in acetonitrile under open circuit conditions for 24 h. The NAB adlayer bonds the exposed carbon surface and attenuates the rate of galvanic corrosion on a joined alloy specimen (lap joint configuration) during a 14-day cyclic accelerated degradation test. The NAB adlayer blocks surface sites, which slows the rate of dissolved oxygen reduction thereby slowing the rate of galvanic corrosion on the nearby alloy. The galvanic corrosion damage on the trivalent chromium process (TCP) conversion-coated alloy was significantly reduced near the composite edges and importantly in the fastener through-hole region. The spontaneously formed NAB adlayer provides better cathodic protection than does the electrochemically formed NAB adlayer, due to more complete coverage with fewer defects (i.e., better blocking layer).

## Graphical abstract

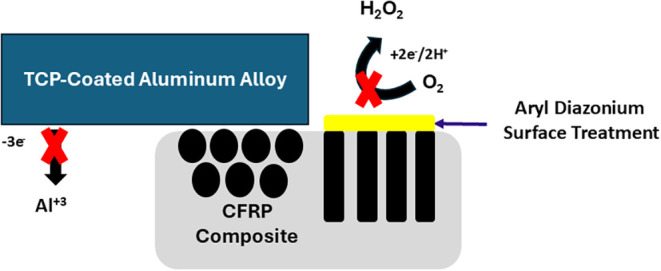

## Introduction

Recent efforts to achieve weight reduction in modern aircraft and automobiles for improved fuel efficiency and reduced carbon dioxide (CO_2_) emissions have led to the extensive use of carbon fiber reinforced polymer (CFRP) composites.^1–8^ Owing to their lightweight, high strength and outstanding fatigue endurance, CFRP composites are used in long-range civilian aircraft, such as the Boeing 787 and Airbus A350, and German cars including BMW and Audi. Carbon fibers in these composites are electrically conductive and more noble than most metals in the galvanic series.^9,10^ CFRP composites are typically used in combination with metal alloys. When using dissimilar material assemblies encompassing composites with exposed carbon fiber surfaces and metal alloys (e.g., aluminum), precautions must be taken to eliminate direct electrical contact between the materials to prevent galvanic corrosion of the nearby metal.^11–14^ The general engineering practice involves electrically isolating the composite from the aluminum alloy using an interlayer of a glass fiber-reinforced composite, a fluorinated polymer film, or a protective coating system.^15–17^ Even with these preventions, severe galvanic corrosion can occur when CFRP composite edges (i.e., exposed carbon fiber surfaces) and aluminum alloys come into direct electrical contact through a condensed layer of atmospheric moisture.^18,19^ In such situations, the more noble carbon fibers act as a cathode and support the electrochemical reduction of dissolved oxygen or protons, depending on the pH of the moisture layer.^16,20,21^ The consumption of electrons in this cathodic reaction accelerates the electro-oxidation or galvanic corrosion rate of the nearby aluminum alloy. Galvanic corrosion can severely degrade the mechanical strength of the aluminum alloy.^13,22^ We believe a viable surface treatment that can be used in combination with common primer and topcoat coatings for mitigating galvanic corrosion at aluminum alloy-CFRP composite joints involves chemically modifying the exposed carbon fiber surfaces with a covalently bonded aryl diazonium adlayer. The adlayer provides cathodic protection by inhibiting the oxygen reduction reaction (ORR) kinetics. This occurs by blocking active sites on the carbon surface for O_2_ chemisorption, which is the initial step in the ORR mechanism. The nm-thick adlayer can also function as a barrier to direct contact with the environment.

A significant body of research exists on the formation of covalently bonded organic adlayers on carbon, metal, and semiconductor surfaces using aryl diazonium salts.^23–33^ The adlayers can be formed using an electrochemically assisted approach or spontaneously by simple immersion. The electrochemical method proceeds initially through the reduction of the aryl diazonium cation in solution to generate an aryl radical at the electrode–solution interface with liberation of N_2_ gas (so-called dediazonation). The radical can then react with the nearby electrode grafting to the surface through a C–C covalent bond. The adlayers can be formed by potential cycling or at fixed potential if the potential excursion is negative enough to drive the 1e^–^ reduction reaction as is shown below (Figure 1) in this well-established mechanism.^23–29^

**Figure 1:**
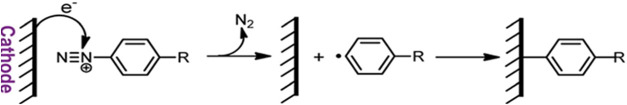
General reaction scheme for the electroduction of an aryl diazonium molecule in solution forming a reactive aryl radical coupled with loss of N_2_.

The adlayers can also be formed spontaneously via electron transfer from the electrode surface to the diazonium molecule in either aqueous or organic solution.^30–33^ Spontaneous grafting involves the immersion of the substrate in an aryl diazonium salt solution under open circuit conditions with no electrochemical induction.^30–33^ For spontaneous deposition on carbon surfaces, the immersion time, reduction potential of the aryldiazonium salt, its concentration, and the choice of solvent will affect the final coverage and morphology of the organic film. While both methods yield adlayers, spontaneous grafting is more practical for real-world application involving large component assemblies or repair processes. We have previously compared electrochemically and spontaneously formed aryl diazonium adlayers on exposed of CFRP composites and demonstrated that the galvanic corrosion rate and material degradation of mechanically joined AA2024-T3 aluminum alloy panels are reduced most by the spontaneously formed 4-nitroazobenzene (NAB) diazonium adlayer (24-h immersion). This was assessed using a legacy neutral salt spray exposure for 14 days (ASTM B117, 5 wt % NaCl, 35 °C) and attributed^34,35^

Reductive electron transfer to the diazonium molecule in solution results in the homolytic cleavage of dinitrogen and the generation of an aryl radical. The radical formed at the electrode–solution interface subsequently bonds to the surface through carbon–carbon and metal–carbon bonds.^23–33,36–41^ Organic adlayer formation resulting from the reduction of a substituted aryl diazonium salt (4-nitrophenyldiazonium tetrafluoroborate in acetonitrile +0.1 M NBu_4_BF_4_) was first demonstrated on glassy carbon in the 1990s by Savéant et al.^23,41^ The authors proposed the formation of an aryl radical from the electroreduction of the diazonium molecule followed by radical attachment to the carbon surface. By choosing different aryl diazonium salts, a wide range of adlayer chemistries can be introduced, allowing for precise tailoring of the electrode surface for specific applications.

In this work, we report on the surface treatment of exposed carbon fiber surfaces in a standard airframe composite with a spontaneously formed adlayer of 4-nitroazobenzene (NAB) diazonium. The surface treatment was evaluated for inhibiting galvanic corrosion on a mechanically joined aluminum alloy 7075-T6 during a cyclic accelerated degradation test. The so-called thin layer mist (TLM, 3.5 wt % NaCl, 55 °C) accelerated degradation test consists of mist wetting, evaporation, salt concentration changes, and heating and cooling cycles during each 24-h period.^42,43^ The results presented herein demonstrate that the galvanic corrosion damage on the aluminum alloy is significantly reduced when the exposed carbon fiber surfaces of the CFRP composite are treated with a spontaneously formed NAB adlayer (24-h immersion). This is attributed to the blocking properties of the adlayer that serve to inhibit the oxygen reduction reaction kinetics and provide a barrier to direct contact with the environment. The spontaneously formed adlayer appears more compact with fewer pinholes and defects than does the comparison electrochemically formed adlayer, and may consist of localized multilayers.^44,45^ The spontaneously formed adlayer is stable during both 14-day neutral salt spray and TLM tests.^35,42^ The results further demonstrate the effectiveness of this surface treatment at reducing the rates of galvanic corrosion and material degradation at composite-alloy lap joints.

## Experimental Methods

### Chemicals and Reagents

Prior to use, the acetonitrile (Sigma-Aldrich) was distilled and stored over activated 5 Å molecular sieves to reduce water impurity. Tetrabutylammonium tetrafluoroborate (NBu_4_BF_4_) was purchased commercially (Sigma-Aldrich) and used without additional purification. The 4-nitroazobenzene diazonium tetrafluoroborate (NAB) was provided courtesy of Professor Richard McCreery and his group at the University of Alberta. Bonderite C-AK 6849 AERO (Henkel Technologies, Madison Heights, MI) was the commercial solution used for degreasing the aluminum alloy specimens. Bonderite C-IC SMUTGO NC AERO (Henkel Technologies, Madison Heights, MI) was the commercial solution used to desmut the aluminum alloy specimens. Bonderite T-5900 RTU (Henkel Technologies, Madison Heights, MI) was the commercial trivalent chromium process (TCP) conversion coating bath used. All aqueous solutions were prepared with ultrapure water (≥18 Ω-cm) from a Barnstead E-Pure water purification system.

### Pretreatment of CFRP Composite Specimens

A standard airframe composite panel (AS4/3501–6) was provided for the research courtesy of the Polymer and Composites Division at the Naval Air Systems Command (Patuxent River, MD). The panel was a unidirectional cross-ply [0/90]­4s laminate prepared with an intermediate modulus polyacrylonitrile (PAN) carbon fiber (AS4 7 μm diam.) and a toughened epoxy prepreg (3501–6) from Hexcel.^9,34,35,42^ The composite specimen thickness was 0.3 cm. Sections cut (2.4 cm × 1.8 cm) from a larger composite piece were used for electrochemical and accelerated degradation testing. It is the cut edges where the exposed carbon fiber surfaces exist. The top and bottom surfaces of the composite specimen are sealed in epoxy so there is very little exposed carbon. For electrochemical testing, electrical connection was made by inserting a copper wire into a hole drilled on one edge of the specimen and affixing it with conducting Ag paste and an epoxy overlayer, as previously described.^9,34,35^ For accelerated degradation testing, a specimen had a 0.6 cm diameter hole drilled near one edge. All three specimen edges, other than the one nearest the hole, were prepared for modification by first abrading for 3 min with 1500 grit aluminum oxide paper wetted with ultrapure water. The 0.6 cm diameter through-hole wall was also pretreated by abrading with the 1500 grit aluminum oxide paper. This was followed by a 10 min ultrasonic cleaning in ultrapure water. Each abraded edge was then polished for 5 min with successively smaller grades of alumina powder (1, 0.3, and 0.05 μm) slurried in ultrapure water on separate felt polishing pads. After each polishing step, the composite was rinsed and ultrasonically cleaned with ultrapure water for 15 min to remove polishing debris. The final ultrasonic cleaning was then performed in purified acetonitrile for 10 min. A specimen used for electrochemical testing received a similar pretreatment, but only on one edge opposite that of the edge where the current collecting Cu wire was affixed.^9,34,35^ For the CFRP composite specimens used in the accelerated degradation testing, a lap joint test specimen was prepared by mechanical joining it with an aluminum alloy plate using a 316L stainless steel fastener, as shown in Figure 2.^34,35^

### Surface Modification of CFRP Composite Specimen Edges

The aryldiazonium salt studied was 4-nitroazobenzenediazonium tetrafluoroborate (NAB). The electrochemically assisted surface modification was performed by cyclic voltammetry.^34,35^ This step involved 25 potential cycles between 0.6 to −0.5 V (vs Ag QRE) in naturally aerated 5 mmol L^–1^ NAB + 0.1 mol L^–1^ NBu_4_BF_4_ in purified acetonitrile at 50 mV s^–1^. The composite specimen was immersed in the solution to near the top edge where the Cu contact wire was positioned such that all three cut edges and the through-hole wall were treated. The spontaneous surface modification was performed under open circuit conditions by immersing the pretreated CFRP composite specimen into a naturally aerated solution of 5 mmol L^–1^ NAB dissolved in purified acetonitrile (no supporting electrolyte). The immersion was performed for 24 h at room temperature as prior work revealed this time produced an effective blocking layer.^34,35^ After both modifications, the composite specimen was thoroughly rinsed with purified acetonitrile to remove any residual NAB molecules and supporting electrolyte.

### Contact Angle Measurements

The wettability of the CFRP composite edge before and after surface treatment was investigated using a static water contact angle measurement. The contact angle for a drop of ultrapure water was measured using a VCA Optima (AST Products, Inc.) video contact angle system. The volume of the water droplet was 0.5 μL. All measurements were made in a dry room with a relative humidity of ≤0.1%.

### Electrochemical Measurements

All the electrochemical measurements were performed using a Reference 600 (Gamry Instruments, Inc.) computer-controlled workstation in a two-compartment, three-electrode glass cell. A platinum (Pt) flag was used as the counter electrode. In nonaqueous solution, a silver wire was employed as the quasi-reference electrode (Ag QRE). In aqueous solution, an Ag/AgCl (3 mol L^–1^ KCl) thermodynamic reference electrode was used. The CFRP composite edge served as the working electrode. Only the composite edge, opposite of that where the current collecting wire was affixed, was utilized in these measurements.^9,34,35^

The electrochemically active surface coverage of NAB was determined by recording cyclic voltammetric *i*–*E* curves from 1.0 to −1.0 V vs Ag QRE to probe the reversible 1-electron oxidation of the pendant NO_2_ groups (NO_2_ + e^–^ ⇄ NO_2_^–•^, *E*_mid_ ∼ −0.75 V vs Ag QRE) in deaerated 0.1 mol L^–1^ NBu_4_BF_4_ and purified acetonitrile. The surface coverage formed by the electrochemically assisted and spontaneous depositions was determined by integrating the charge for the oxidation reaction (NO_2_^–•^ → NO_2_ + e^–^) peak current using eq 1

1 Q=nFAΓ

in which *Q* is the charge (C), *n* is the number of electrons transferred per molecule, *F* is the Faraday constant (96,485 C mol^–1^), *A* is the composite specimen edge geometric area (cm^2^), and Γ is the surface coverage (mol cm^–2^). Deaeration was performed by purging N_2_ gas through the solution for 20 min.

### Preparation of the Aluminum Alloy (AA7075-T6) Specimens

Commercial wrought aluminum alloy 7075-T6 was obtained as a 1 mm-thick sheet (www.onlinemetals.com) and cut into 5.4 cm × 3.0 cm pieces (18.9 cm^2^). The alloy specimen was mechanically abraded with wet P1500 grit aluminum oxide grinding paper for 4 min followed by ultrasonic cleaning in ultrapure water for 20 min. The specimen was then fine polished with 0.3 μm alumina powder (Buehler) slurried in ultrapure water on a felt polishing pad for 4 min and ultrasonically cleaned in ultrapure water for another 20 min to remove polishing debris. This was followed by a degreasing step at 55 °C for 10 min in 20% v/v Bonderite C-AK 6849 AERO alkaline degreaser. Following this, the specimen was gently rinsed with flowing city tap water for 2 min. The specimen was then desmutted by immersion in 20% v/v Bonderite C-IC SMUTGO NC AERO at room temperature for 2 min. This was followed by another gentle 2-min tap water rinse. The alloy specimen was then surface treated with a trivalent chromium process (TCP) conversion coating by immersion in Bonderite T5900 Ready-to-Use (Henkel Technologies) at room temperature for 10 min. Finally, the TCP-coated specimen was rinsed by immersion, with periodic agitation, in a beaker of city tap water for 2 min, and then in a beaker of ultrapure water for 30 s. The specimen was then dried and aged in the laboratory overnight in a covered dish before further use.

### Configuration of the CFRP Composite and AA7075-T6 Specimens in Lap Joint Test Specimens

A TCP-coated AA7075-T6 specimen was mechanically joined with an unmodified or a NAB-modified CFRP composite specimen using a stainless-steel fastener (SS316L), as presented in Figure 2.^34,35,42^ The mechanical fastener was tightened to a torque of 45 lb-in. The stainless-steel bolt threads were wrapped with Teflon tape to minimize direct electrical contact with the composite and aluminum alloy through-hole walls. The bolt head and nut were wet sealed with a commercial silicon sealant (DAP KWIK SEAL ULTRA) to repel water and to electrically isolate the metal from the solution mist. The back side of each aluminum alloy specimen was covered with corrosion protection tape (Scotchrap, 3 M Company) so that only the front surface of the alloy was in direct contact with the composite and exposed during the accelerated degradation testing.

**Figure 2:**
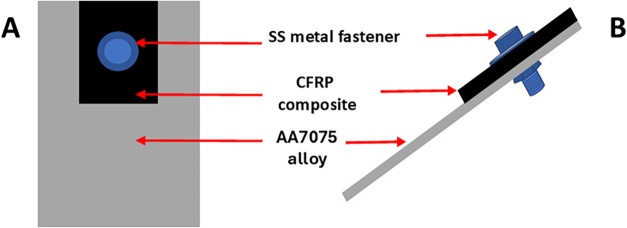
Configuration of the lap joint aluminum alloy-CFRP composite specimen used in the accelerated degradation testing. (A) Top view. (B) Side view. Dimensions: CFRP composite (2.5 × 1.8 × 0.3 cm^3^) and AA7075-T6 alloy panel (5.4 × 3.0 × 0.1 cm^3^). The exposed carbon fibers in the composite are along the cut edges and in the through-hole wall. SS = 316L stainless-steel fastener.

As mentioned above, a CFRP composite specimen was cut into the following dimensions, 2.5 × 1.8 cm^2^. The composite thickness was 0.3 cm. A fastener hole, 0.6 cm in diameter, was drilled through both the metal and composite. The large area top and bottom surfaces of the composite were covered by the epoxy matrix so the carbon fibers in these regions are electrically and electrochemically isolated. Therefore, only the carbon fibers on the cut edges and in the through-hole wall were exposed. The three cut edges and the through-hole region were surface treated with the NAB adlayer by either the electrochemical or spontaneous adlayer formation process. The total geometric area of carbon fibers exposed on the three cut edges and the through-hole wall was 3.15 cm^2^. This resulted in a cathode-to-anode exposed area ratio of 3.15 cm^2^/18.9 cm^2^ or 0.17 in the lap joint test specimens.

### Neutral Salt Spray (NSS) Accelerated Degradation Test (ASTM B117)

Some comparison tests were made with the lap joint specimens exposed to a standard neutral salt spray similar to testing we have reported on previously.^35,42,43^ Mechanically fastened aluminum alloy-CFRP composite lap joint specimens were placed at a ∼20° angle (with respect to the vertical axis) on plastic racks inside a commercial salt spray chamber (Associated Environmental Systems-MX 9204). The bolted end of the specimens was highest on the rack. The test specimens were exposed to a continuous salt fog generated from 5 wt % (0.86 mol L^–1^) NaCl at 35 ± 1 °C for 14 days according to ASTM B117 (Standard Practice for Operating Salt Spray (Fog) Apparatus). At the end of the 14-day test period, the test specimens were removed, disassembled, and rinsed with and ultrasonically cleaned in ultrapure water for 30 min to remove salt deposits. The alloy specimens were then ultrasonically cleaned in concentrated HNO_3_ for 10-min periods to dissolve corrosion product, dried thoroughly with N_2_ gas, and weighed. Ultrasonic cleaning in HNO_3_ was repeated until a constant weight was achieved.^46^ The mass loss of the aluminum alloy over the 14-day period provided a measure of the galvanic corrosion rate.

### Thin Layer Mist (TLM) Accelerated Degradation Test

For this cyclic accelerated degradation test, aluminum alloy-CFRP composite lap joint specimens were positioned horizontally on a platform above an ultrapure water layer inside a loosely sealed polypropylene container, as shown in Figure 3.^42,43^ Separate containers were used for each specimen. The test involved the following steps:

(i) Each specimen was misted with one spray (∼1 ft. distance) of a 3.5 wt % NaCl solution from a nebulizing spray bottle at room temperature and placed horizontally in a polypropylene container above a layer of water.^42,43^ The loosely sealed container was then placed in an oven at 55 °C for 23 h. The water at the bottom of the container ensured that the relative humidity inside the container remained near 100% during the test. The layer of mist consisted of water droplets as small in size and with as uniform a diamater as was possible given the application method.
(ii) At the end of a 23-h period at temperature, the container was removed from the oven, opened, and the specimen allowed to cool to room temperature in the laboratory air for 30 min.
(iii) The specimen was then remisted with one spray of the 3.5 wt % NaCl solution. The container remained open to the atmosphere for ∼30 min after applying mist before being loosely sealed and placed back in the oven at 55 °C for another 23-h period of constant temperature heating.

**Figure 3:**
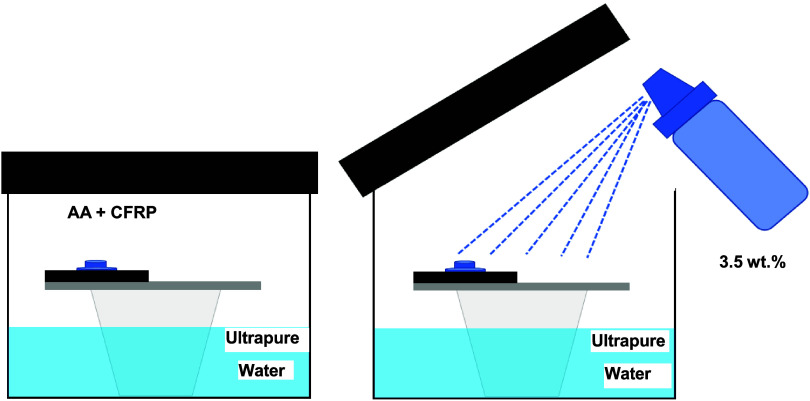
A schematic diagram of the thin layer mist (TLM) test configuration and specimen positioning.

One full test cycle was 24 h during which time the specimen surface experienced mist droplet wetting, evaporation, and a local salt concentration increase, but did not completely go to dryness because of the water vapor in the container. The entire test period was 14 cycles or days. At the end of the 14-day test period, the lab joint test specimens were removed, disassembled, and rinsed with and ultrasonically cleaned in ultrapure water for 30 min to remove salt deposits. The accumulated corrosion product on the alloy specimens was then removed by ultrasonic cleaning in concentrated HNO_3_ for 10-min periods followed by thorough rinsing with ultrapure water and drying under a stream of N_2_ gas. Ultrasonic cleaning in HNO_3_ was repeated until the weight loss of a specimen was negligible (≤0.002 g).^46^

### Evaluation of Metal Alloy Corrosion Damage

The corrosion damage on each AA7075-T6 panel was assessed both qualitatively and quantitatively. The surface topography of corrosion damage sites on an acid cleaned AA7075-T6 panel was evaluated using a Keyence VHX 6000 digital optical microscope. Surface roughness (*S*_*q*_) and peak-to-valley heights (*S*_*z*_) were calculated from the 3D topographical profiles. *S*_*q*_ (μm) is the root-mean-square of the height features and reflects the standard deviation of the feature heights. *S*_*z*_ (*S*_*p*_ + *S*_*v*_, μm) is the sum of the largest peak height (*S*_*p*_) and largest pit depth (*S*_*v*_). For higher resolution imaging, a JEOL 6610LV scanning electron microscope housed at the MSU Center for Advanced Microscopy was used. The mass of the entire TCP-coated aluminum alloy panel was measured before and after the accelerated degradation test to determine the mass change (loss). The corrosion intensity (CI) was then calculated from this mass loss (*g*) using eq 2

2 $$\mathrm{CI}=\frac{g_{\mathrm{loss}}}{m^{2}y}$$

in which *m* is the exposed area of the alloy panel (*m* ^2^) and *y* is time in years calculated as $y=14\;days\times \frac{1\;y}{365\;days}$ Since the backside of the TCP-coated alloy panel was covered in corrosion protection tape during the test and only the front side of the panel was exposed to the salt spray or thin layer mist, the area (*m* ^2^) used in the calculation of the CI was the geometric area of the front surface less the area of the through-hole.

The following scale, developed by the U.S. Army Material Command, was utilized to grade the corrosion damage on an aluminum alloy panel after the 14-day NSS and TLM exposures.^47^ The grading scale is as follows: Stage 0—no visible corrosion, Stage 1—simple discoloration and staining, Stage 2—loose rust or corrosion product and early stage pitting along with minor etching, Stage 3—rust or corrosion product, minor etching, pitting, and more extensive surface damage, and Stage 4—significant rust or corrosion product formation, extensive etching, blistering, and pitting that has progressed to the point where the life of the specimen has been affected.^47^

## Results

### Surface Modification

The surface chemistry of carbon materials, such as diamond, glassy carbon, carbon nanotubes and graphene, and metals (Au, Pt, Ni) can be controlled by bonding aryl diazonium (^+^N_2_–Ar-R) molecules.^23–41^ Reductive electron transfer to an aryl diazonium molecule in solution results in the homolytic cleavage of the ^+^N_2_–Ar bond producing dinitrogen and an aryl radical. The radical is formed at the electrode–solution interface and subsequently bonds to the surface through carbon–carbon and metal–carbon bonds.^23–27,36–41^

The spontaneous grafting of an NAB adlayer on the exposed carbon fiber surfaces of a composite was achieved by immersing the entire specimen (except for the top edge with the Cu wire) in 5 mmol L^–1^ NAB dissolved in purified acetonitrile under open circuit conditions for 24 h. This ensured that all cut edges and the fastener through-hole wall were modified. This immersion time was used because prior published voltammetric studies indicated the oxygen reduction reaction current is suppressed up to 99% by an NAB adlayer formed spontaneously for 24 h.^34,35^ In other words, 24 h period appears required to form a complete, low defect adlayer on the carbon surface. The successful attachment of NAB admolecules on the exposed carbon fibers in the absence of electrochemical assistance was confirmed by Raman spectroscopy, as described in prior published work.^35^ Evidence of the NAB grafting in this work was confirmed by probing the electrochemical activity of the pendant aryl-NO_2_ functional groups. Figure 4 shows a cyclic voltammetric *i*–*E* curve for a modified CFRP composite edge (edge opposite that where the current collecting Cu wire was affixed) in a deaerated acetonitrile containing 0.1 mol L^–1^ NBu_4_BF_4_. The composite edge was contacted with the electrolyte solution via a convex meniscus.^9,34,35^ Reduction and oxidation peaks are observed at ca. −0.9 and −0.7 V vs AgQRE, respectively, that correspond to the reversible 1e^–^ reduction of the nitro (-NO_2_) group on the grafted NAB admolecules.^24,27,33–36,39,42,48^

**Figure 4:**
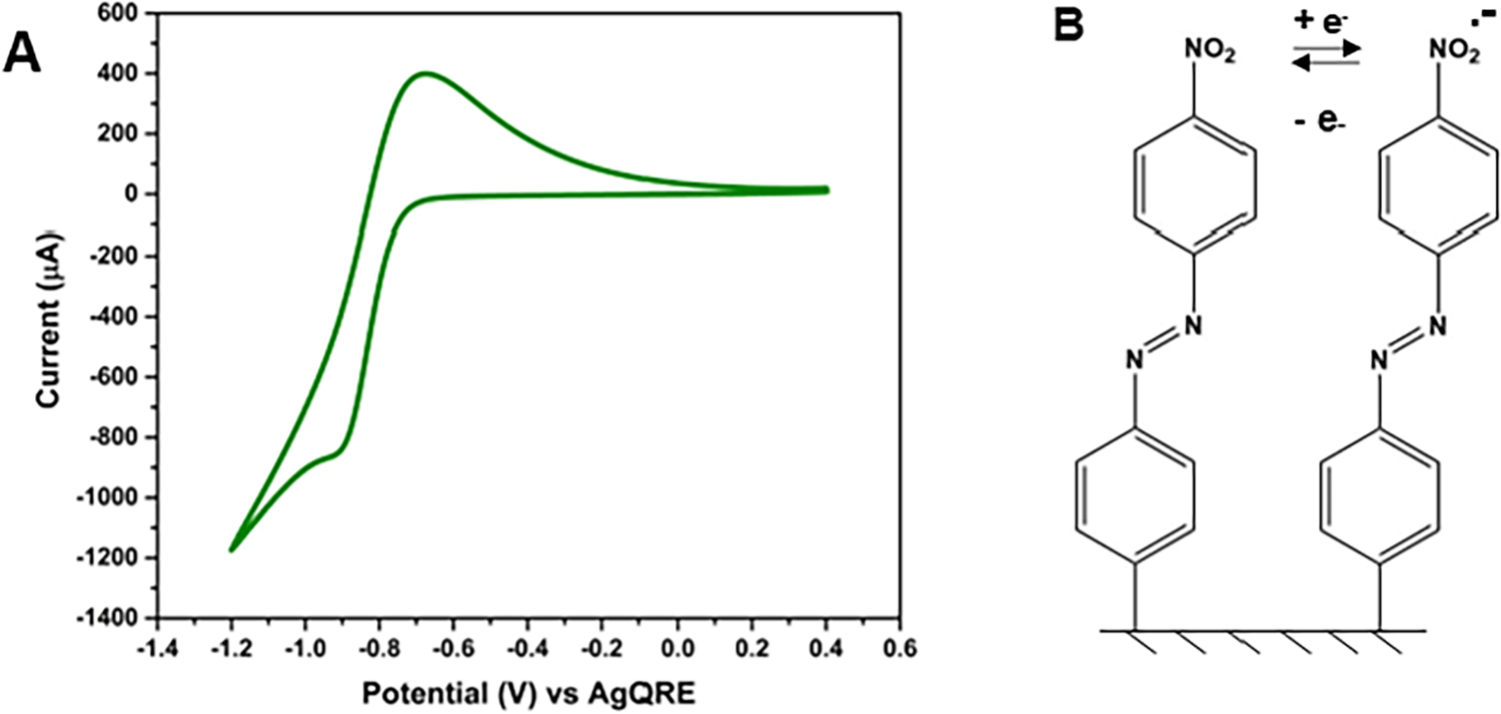
(A) Cyclic voltammetric *i*–*E* curve for a spontaneously modified CFRP composite edge recorded in a deaerated and purified acetonitrile solution containing 0.1 mol L^–1^ NBu_4_BF_4_. The scan rate was 50 mV s^–1^. Grafting was performed by immersing the composite in a naturally aerated solution of acetonitrile containing 5 mmol L^–1^ NAB for 24 h. (B) Illustrates the reversible 1e^–^ redox reaction of the pendant NO_2_ functional group on the NAB admolecule.

The reduction of the NO_2_ group occurs concomitant with an additional cathodic current that is attributed to the reduction of some water impurity. This water impurity likely originated from the hydroscopic NBu_4_BF_4_ salt that was not dried prior to use. Integrating the oxidation peak charge, as it is devoid of charge from this secondary parasitic redox reaction, was used to estimate the electrochemically active surface coverage according to eq 1. A nominal surface coverage of 4.3 ± 0.4 nmol cm^–2^ was determined; a nominal value consistent with the coverage reported previously for an electrochemically formed 4-NAB adlayer of 3.1 ± 1.1 nmol cm^–2^.^35^ The surface coverage is reported as mean ± std. dev. for three different composite specimens. The data are normalized to the geometric area of the composite edge. It should be noted that the surface coverage calculation by cyclic voltammetry only considers the electrochemically active admolecules on the electrode surface. Therefore, the true surface coverage may be higher. The theoretical coverage for a monolayer of close-packed NAB admolecules on a flat surface, assuming end-on bonding through the phenyl ring, is 1.25 nmol cm^–2^.^48,49^ The 3× larger surface coverage determined for the spontaneously modified composites may reflect multilayer formation at least in some regions. There is one point of caution when using this nonaqueous voltammetric measurement to estimate the electrochemically active surface coverage. The reversible reduction of dissolved oxygen (O_2_/O_2_^•–^) turns out to occur at the same potentials as the aryl NO_2_/NO_2_^•–^ redox reaction. While the voltammetric measurements were performed herein under deaerated conditions, any residual O_2_ can result in an overestimate of the charge and therefore the surface coverage.

Figure 5 presents photographs of ultrapure water droplets on (A) unmodified and (B) spontaneously modified (NAB 24 h immersion) CFRP composite edges. The nominal static contact angle on the unmodified composite edge was 57.3 ± 1.6° (*n* = 3 different composites). The nominal contact angle on the spontaneously modified composite edge was higher at 74.5 ± 2.6° (*n* = 3 different composites). The higher contact angle is attributed to the reduced wettability and increased hydrophobicity of the NAB modified carbon surface. Recall that the carbon fibers are embedded in epoxy so the contact angle magnitude is affected by both the chemical properties of the NAB adlayer as well as the epoxy. Even so, the increased contact angle is consistent with increased surface hydrophobicity due to the presence of the NAB adlayer.

**Figure 5:**
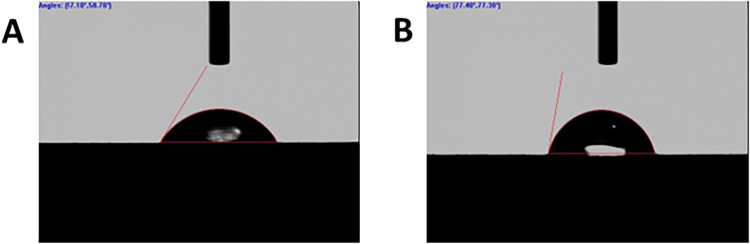
Photographs of water droplets and static contact angles for CFRP composite edges (A) unmodified and (B) spontaneously modified with NAB. The composite was spontaneously modified by immersion in naturally aerated 5 mmol L^–1^ NAB dissolved in purified acetonitrile under open circuit conditions for 24 h.

### Thin Layer Mist (TLM) Accelerated Degradation Test

Figure 6 shows digital optical micrographs of TCP-coated AA7075-T6 alloy panels before and after the 14-day TLM test. The lap joint test configuration shown in Figure 1 was used with the TCP-coated alloys joined to either an unmodified or spontaneously modified composite. Figure 6A shows a TCP-coated alloy specimen prior to testing. There are no visible defects, surface imperfections, or discolorations prior to the test anywhere on the panel. Figure 6B,C show TCP-coated alloy specimens after the 14-day TLM test and removal of the composite. The micrographs reveal distinctly different galvanic corrosion damage patterns for alloy panels joined with unmodified (Figure 6B) and spontaneously modified (Figure 6C) composite specimens. On both panels, a rectangular outline can be seen indicating the position of the composite. The region within this area was underneath the composite during the test, including the fastener through-hole. Figure 6B reveals the TCP-coated alloy panel coupled to the unmodified composite is extensively corroded. The corroded regions appear tan in the micrograph and are localized in the regions along the composite edges, underneath the composite, and around the through-hole. The red arrows indicate the discolored region on the alloy that was near a composite edge during the test. The discoloration is present around all three sides of the composite and reflects sites where metal dissolution has occurred. The corrosion damage extends outward from the composite edges by ca. 5 mm. This is corrosion damage is indicated by the green arrows. The blue arrows indicate sites of crevice corrosion damage (light tan regions) on the alloy panel underneath the composite and near the through-hole.

**Figure 6:**
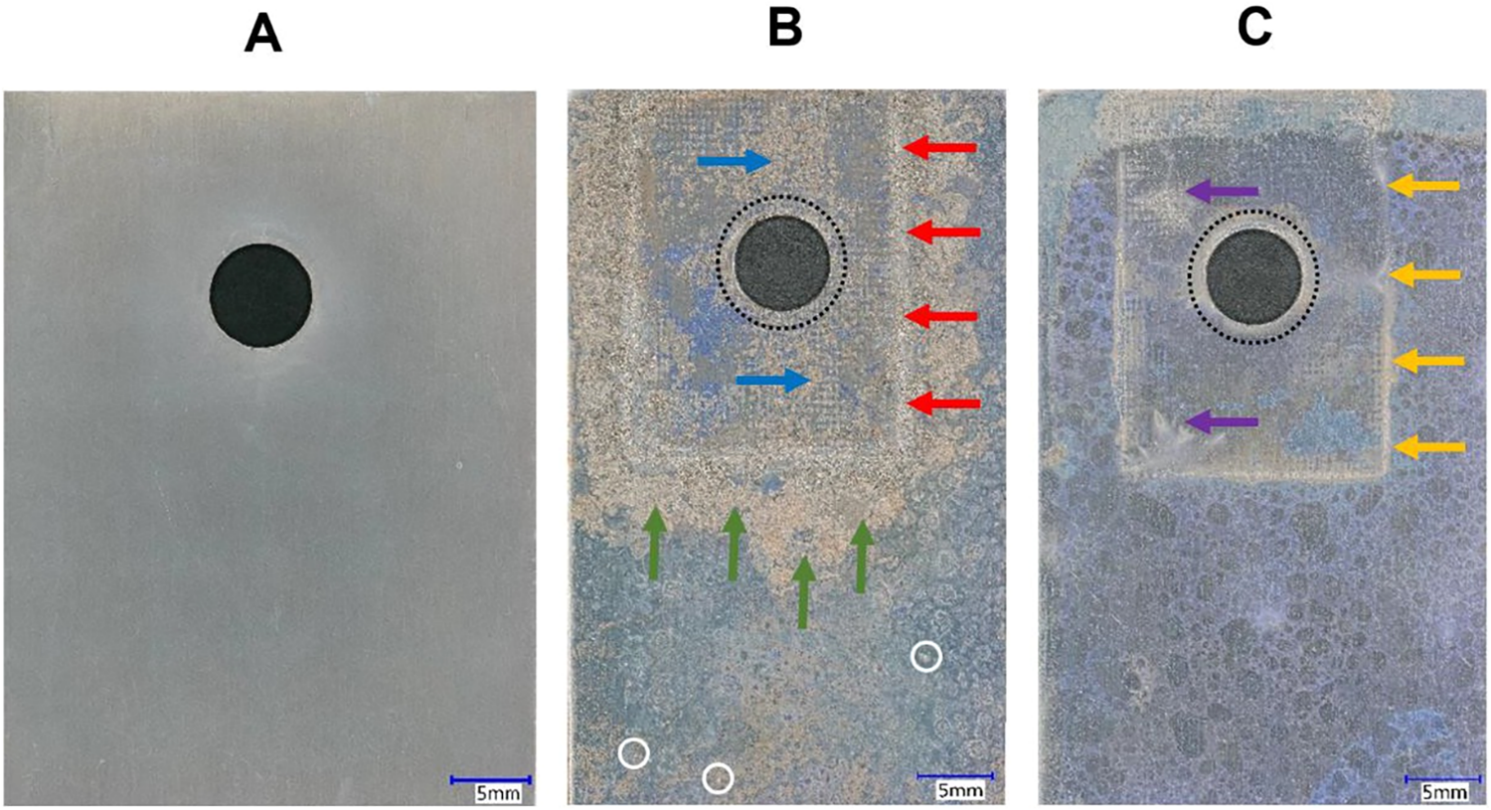
Digital optical micrographs of TCP-coated AA7075-T6 panels (A) before testing, (B) joined with an unmodified composite and (C) joined with a spontaneously modified (NAB, 24 h immersion) CFRP composite after 14 days of TLM exposure. The micrographs show the entire panel and were collected with a 30× objective.

Figure 6C shows the TCP-coated alloy specimen joined with a composite spontaneously modified with NAB (24 h immersion) has less visible corrosion damage. The yellow arrows indicate the discolored regions near the composite edge during the test. The discoloration is present around all three sides of the composite. However, unlike the panel joined with the unmodified composite, there is much less metal dissolution. There is less corrosion away from the composite edges outward on the panel and there is qualitatively less crevice corrosion damage underneath the composite and around the through-hole. Two regions of metal damage under the composite seen are demarked with the purple arrows.

The spontaneous modification of the CFRP composite by simple immersion for 24 h inhibits the oxygen reduction reaction rate, as has been demonstrated in prior research,^34,35,42^ and thereby attenuates the galvanic corrosion rate on the nearby TCP-coated alloy panel. Additionally, the crevice corrosion underneath the composite is reduced. We suppose this results because of a tighter seal between the composite and the alloy panel. The immersion in the acetonitrile softens the hard epoxy layer on the composite making it a little tacky such that a tight seal results when it is fastened with the metal.

The digital micrographs in the preceding figure provide only a qualitative indication of corrosion damage across the alloy surface. Topographical analysis provides more objective evidence for the benefit of the spontaneously formed NAB adlayer in terms of inhibiting galvanic corrosion. Figure 7A shows three representative regions on a TCP-coated aluminum alloy panel where topographical (*x*–*z*) line scans were recorded across the discoloration around the edges of a composite (red bars). Each line profile was ca. 8 mm in length starting at a location under the composite, crossing the discolored region near the composite edge seen in Figure 6B,C, and extending out further on the panel. Figure 7B shows representative line scan profiles from the left, bottom and right sides of two TCP-coated alloy panels, one that was joined with an unmodified and one that was joined with a spontaneously modified composite after the 14-day TLM test. The line profiles for the TCP-coated panel joined with an unmodified composite exhibit a significant amount of metal dissolution starting from the edge of the composite. The metal dissolution extends deep into the alloy (50–100 μm) and outward from the edge by 1000–3000 μm (1–3 mm) undercutting the TCP coating. The galvanic corrosion damage progresses into the alloy panel near a composite edge with a nominal peak-to-valley height of 113 ± 23 μm across the line profiles. In contrast, there is little to no metal dissolution on the panel joined with spontaneously modified CFRP composite specimen. The peak-to-valley height across the line profiles was 31 ± 5 μm. The surface is rougher than is a TCP-coated alloy before TLM exposure, which is 4.1 ± 0.7 μm, but there is no large-scale metal dissolution. These line profiles confirm that the discoloration seen in Figure 6B is actually regions of significant metal dissolution, while the discoloration in Figure 6C is with minor surface roughening.

**Figure 7:**
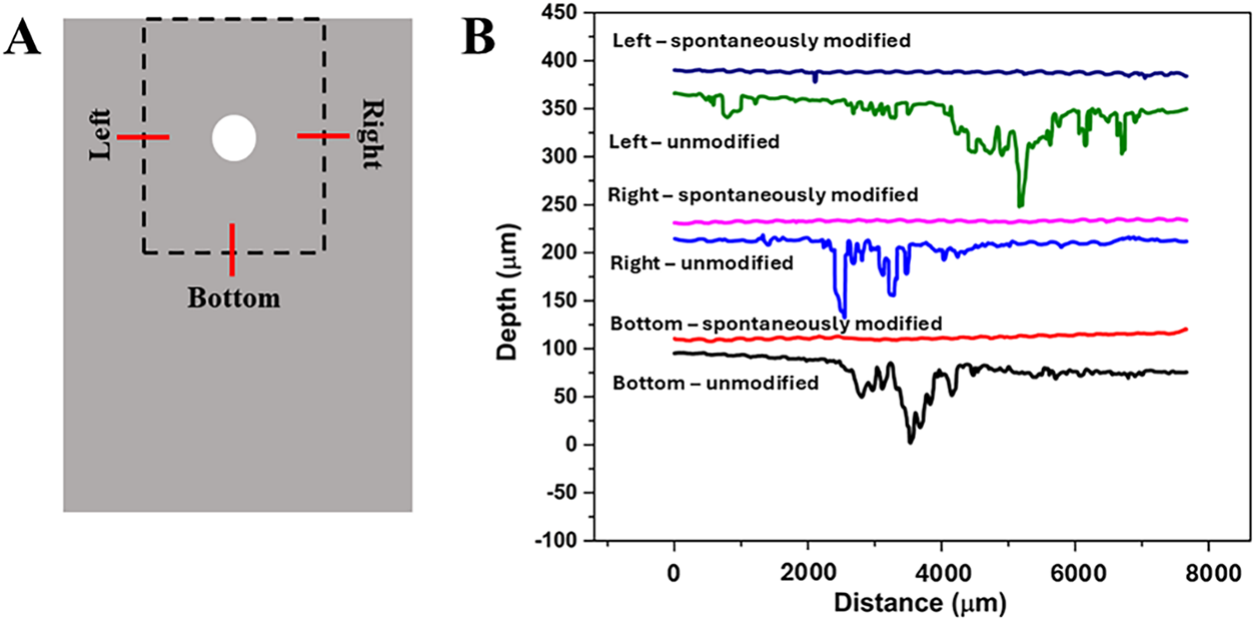
Digital optical microscopy surface topography line profiles (*x*–*z*) after the 14-day TLM test revealing the depth and lateral expansion of corrosion damage on TCP-coated AA7075-T6 panels in the regions adjacent to where the composite was positioned during the accelerated degradation test. (A) Schematic diagram showing regions on each side of a position composite where the line profiles were recorded. (B) Surface topography line profiles on TCP-coated AA7075-T6 panels joined with an unmodified and a spontaneously modified CFRP composite (NAB, 24-h immersion) after the 14-day TLM test. The micrographs were recorded at a 500× magnification.

On aircraft, a critical galvanic damage location is in and around the fastener through-holes used to join the composite to an alloy. These sites are buried in assemblies with protective coatings (primer and paint) making visible detection of early stage corrosion damage difficult. Not only does the NAB adlayer spontaneously formed on the exposed composite edges inhibit galvanic corrosion on the nearby TCP-coated alloy, but it functions effectively to inhibit through-hole corrosion. Figure 8 shows SEM micrographs of fastener through-hole regions on TCP-coated alloy panels after the 14-day TLM test. Micrographs are presented for an alloy joined with an unmodified composite and one joined with a composite spontaneously modified with NAB for 24 h. The area around the hole on the TCP-coated alloy surface joined with an unmodified composite is presented in Figure 8A. Significant metal dissolution is present on the panel surface near the hole, as indicated by yellow arrows. This area was underneath the composite during the test. In contrast, Figure 8B reveals much less corrosion damage near the hole on the alloy surface that was joined with a composite spontaneously modified with NAB (24 h). Two areas of very minor damage are indicated by green arrows. The TCP-coated alloy panel joined to the spontaneously modified composite exhibits Stage 1 damage in the form of simple discoloration and staining, whereas the alloy panel joined to the unmodified composite shows Stage 3 damage with more extensive metal dissolution.

**Figure 8:**
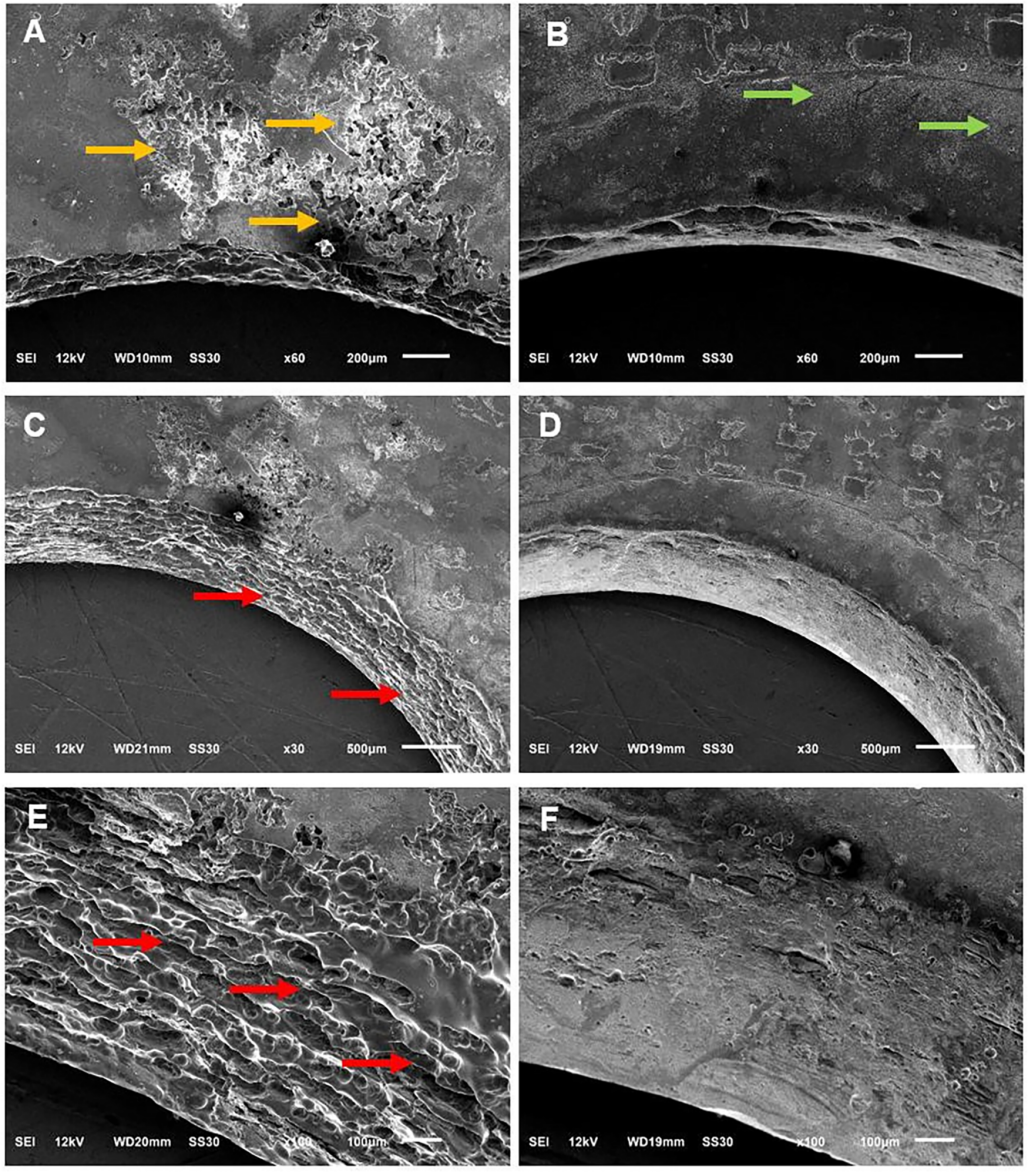
SEM micrographs of fastener through-holes on TCP-coated AA7075-T6 alloys after the 14-day TLM test. (A) a top view of the fastener hole on the surface of the alloy panel joined to an unmodified CFRP composite, (B) a top view of the hole on the surface of the alloy panel joined to a spontaneously modified composite, (C) the hole wall of the alloy joined to an unmodified composite at a 28° tilt angle, (D) the hole wall of the alloy joined to a spontaneously modified composite at a 28° tilt angle, (E) the hole wall of the alloy joined to an unmodified composite at a 40° tilt angle, and (F) the hole wall of the alloy joined to a spontaneously modified composite at a 40° tilt angle.

During the TLM test, the TCP-coated alloy and composite specimen were joined together using a stainless-steel fastener with the threads wrapped with Teflon tape to minimize direct electrical contact with the alloy or composite. Additionally, the fastener head and washer/nut were covered with silicone sealant to inhibit moisture penetration and prevent direct contact with the environment. Even with these protections in place, the alloy hole walls showed signs of degradation after the test, particularly for the alloy panel joined to an unmodified composite. This can be seen by comparing the micrographs in Figure 8C,D. The micrographs were recorded at a 28° specimen tilt angle. There is extensive pitting corrosion damage, as indicated by red arrows, on the alloy joined with an unmodified composite. In contrast, there is negligible hole wall corrosion damage on the alloy joined to the spontaneously modified composite as the surface is much smoother resembling that of the hole before testing. Figure 8E,F present SEM micrographs of the hole wall on the two panels at an even greater tilt angle of 40° to better reveal the corrosion damage or lack thereof. The NAB surface treatment of the composite hole wall significantly reduces the galvanic corrosion in and around the fastener through-hole of the aluminum alloy. In application, this surface treatment would be used in combination with typical surface coatings and finishes (primer and paint layers) and serve as a last line of defense against galvanic corrosion when coating breakthrough happens near a joint.

Weight loss and corrosion intensity (CI) data for TCP-coated alloy panels joined with unmodified and spontaneously modified (NAB) composites after the 14-day TLM test are presented in Figure 9. These data provide a more quantitative assessment of the corrosion inhibition provided by the spontaneously formed NAB adlayer. The TCP-coated alloy joined with a NAB-modified composite exhibited weight loss and corrosion intensity values 84% lower than the values for the TCP-coated alloy joined with the modified composite after this cyclic test.

**Figure 9:**
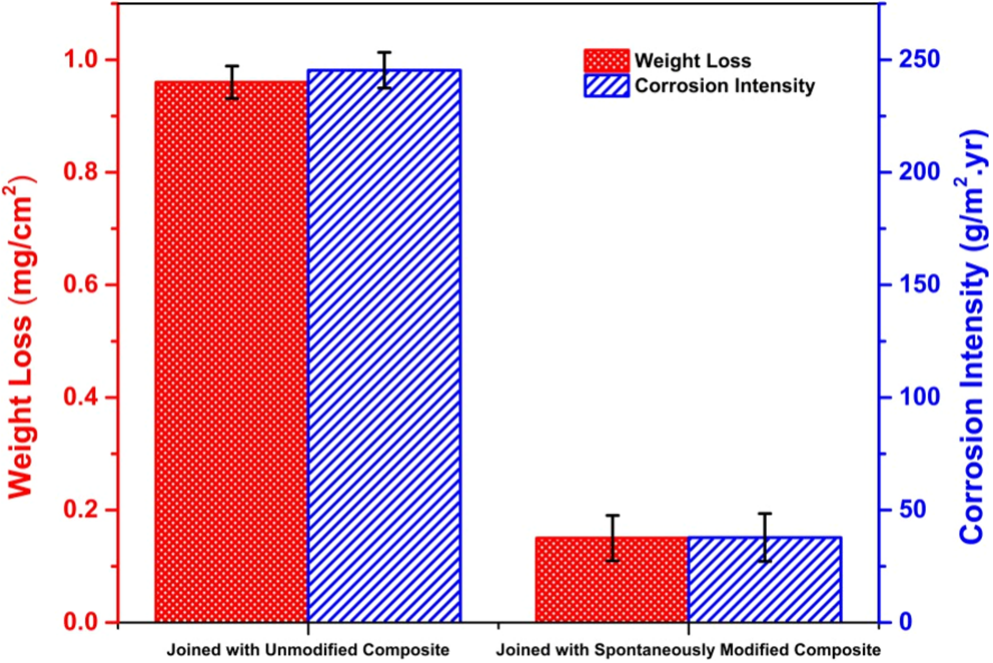
Weight loss and corrosion intensity for TCP-coated AA7075-T6 panels joined with unmodified and spontaneously modified (NAB, 24-h immersion) CFRP composites after 14 days of TLM exposure. Data are presented as mean ± std. dev. for *n* = 3 specimens of each type.

### Neutral Salt Spray (NSS) Accelerated Degradation Test

For additional evidence of the effectiveness of the surface treatment, we investigated the degree to which galvanic corrosion damage on uncoated and TCP-coated AA7075-T6 panels is reduced when mechanically joined to a CFRP composite unmodified and modified with a NAB adlayer during a 14-day legacy neutral salt spray exposure. Composite specimens were treated with a NAB adlayer formed electrochemically (25 cycles) and spontaneously (24-h immersion), for comparison. Lap joint specimens were then exposed to a continuous neutral salt spray (NSS) of 5 wt % NaCl at 35 ± 1 °C for 14 days.

The findings are consistent results reported in our prior publication for aluminum alloy 2024-T3 joined with CFRP composite specimens surface treated with NAB.^34,35^ A series of digital optical micrographs of different AA7075-T6 test panels before and after the NSS exposure are presented in Figure 10. The TCP-coated alloy prior to testing (Figure 10A) is smooth and featureless. There is no discoloration anywhere on the panel surface. The uncoated alloy panels joined with an unmodified CFRP composite (Figure 10B,C) are characterized by widespread corrosion damage near and away from the fastener hole. The damage includes metal dissolution underneath where the composite was joined (crevice corrosion), near where the composite edge was positioned (trenching or galvanic corrosion), and away from where the composite was joined (localized pitting). Importantly, there is major corrosion damage in and around the fastener hole. The corrosion damage (Stage 3–4) is extensive after just 3 days (Figure 10B) and becomes more severe and widespread after 14 days (Figure 10C).

**Figure 10:**
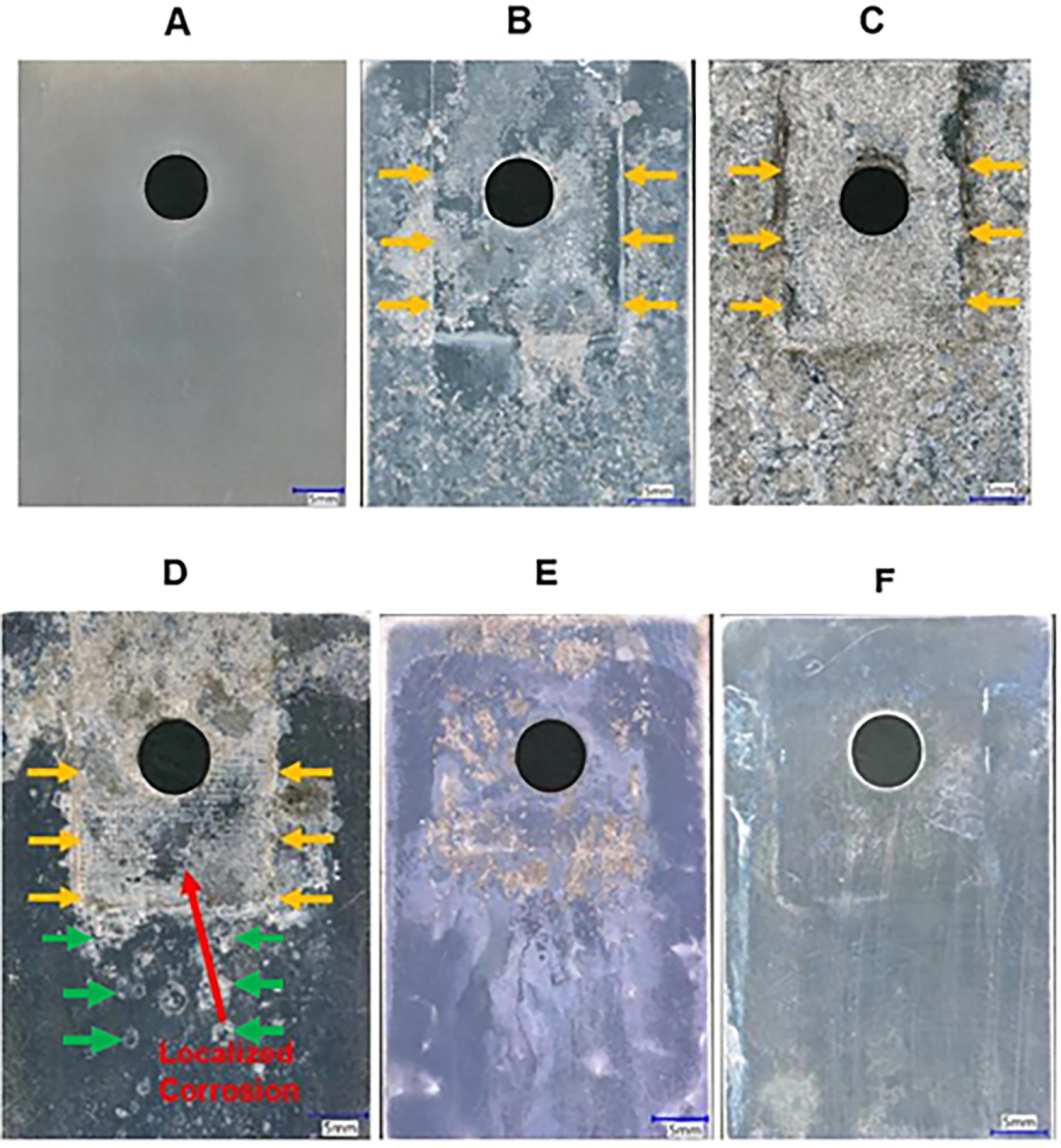
Digital optical micrographs of AA7075-T6 panels. (A) TCP-coated and before neutral salt spray (NSS) exposure, (B) uncoated alloy and joined with an unmodified CFRP composite after just 3 days of NSS exposure, (C) uncoated alloy and joined with an unmodified CFRP composite after 14 days of NSS exposure, (D) TCP-coated and joined to an unmodified composite after 14 days of NSS exposure, (E) TCP-coated and joined to an electrochemically modified composite after 14 days of NSS exposure, and (F) TCP-coated with a spontaneously modified CFRP composite after 14 days of NSS spray exposure at 30×. The scale bar in each micrograph is 5 mm.

For the TCP-coated AA7075-T6 panel (Figure 10D) joined to unmodified CFRP composite, the TCP coating inhibits corrosion on the alloy panel away from the composite (green arrows) during the 14-day test. Prior work has revealed the TCP conversion coating protects aluminum alloys from corrosion through both anodic and cathodic inhibitory mechanisms.^43,50–58^ The composition of AA7075-T6 includes the following alloying elements (wt %): 5.1%–6.1% Zn, 2.1%–2.9% Mg, 1.2–2.0% Cu and less than 0.5% of Si, Fe, Mn, Ti, Cr, etc.^57^ The TCP coating (∼200 nm thick) provides cathodic protection by blocking sites on the cathodic Fe- and Cu-rich intermetallic sites where dissolved oxygen reduction occurs at a relatively high rate.^43,50–58^ The anodic protection is provided on the aluminum matrix with the coating serving as a barrier layer that reduces contact with the environment, thereby slowing the metal oxidation reaction rate.^43,50–58^ There is still significant damage on the TCP-coated near the composite edges, underneath the composite, and in and around the fastener through hole when the CFRP composite is untreated as indicated by the yellow arrows.

In contrast, the micrographs in Figure 10E,F reveal significantly reduced corrosion damage on the TCP-coated aluminum alloy near the composite edges and underneath the composite when the exposed carbon fibers of the composite were surface treated with a NAB adlayer. The TCP-coated AA7075-T6 panel joined to a CFRP composite electrochemically modified with NAB (Figure 10E) exhibits far less damage along the perimeter of the composite position and underneath the composite (Stage 2) as compared to the TCP-coated AA7075-T6 panel joined to an unmodified composite (Figure 10D). There is some discoloration on the panel away from where the composite was joined. This may be due to some localized corrosion at TCP coating imperfections. Notably, there is even less visible damage on the TCP-coated alloy panel joined to a CFRP composite spontaneously modified with NAB for 24 h (Figure 10F). Only minor discoloration and staining (Stage 1) are observed. In this test paradigm, the 24-h spontaneously formed NAB adlayer is qualitatively more effective than the electrochemically formed adlayer at inhibiting galvanic corrosion on the TCP-coated alloy. This attenuation results from the decreased rate of O_2_ reduction on the exposed carbon fibers, as has previously been discussed.^34,35,42^

The galvanic corrosion damage was assessed more quantitatively using 3D digital microscopy. The topographical analysis was performed at five different spots on each alloy panel in the region adjacent to where the composite was joined (i.e., areas of greatest galvanic corrosion damage). The area analyzed per spot was 1000 μm × 1000 μm at a 500× magnification. A schematic diagram is presented in Figure 11A showing the regions on each panel where the 3D topographic profiling was performed. Figure 11B presents surface roughness (*S*_*q*_) and peak-to-valley height (*S*_*z*_) data for TCP-coated AA7075-T6 panels joined with CFRP composites surfaces unmodified, electrochemically modified, and spontaneously modified with NAB after 14 days of NSS exposure.

**Figure 11:**
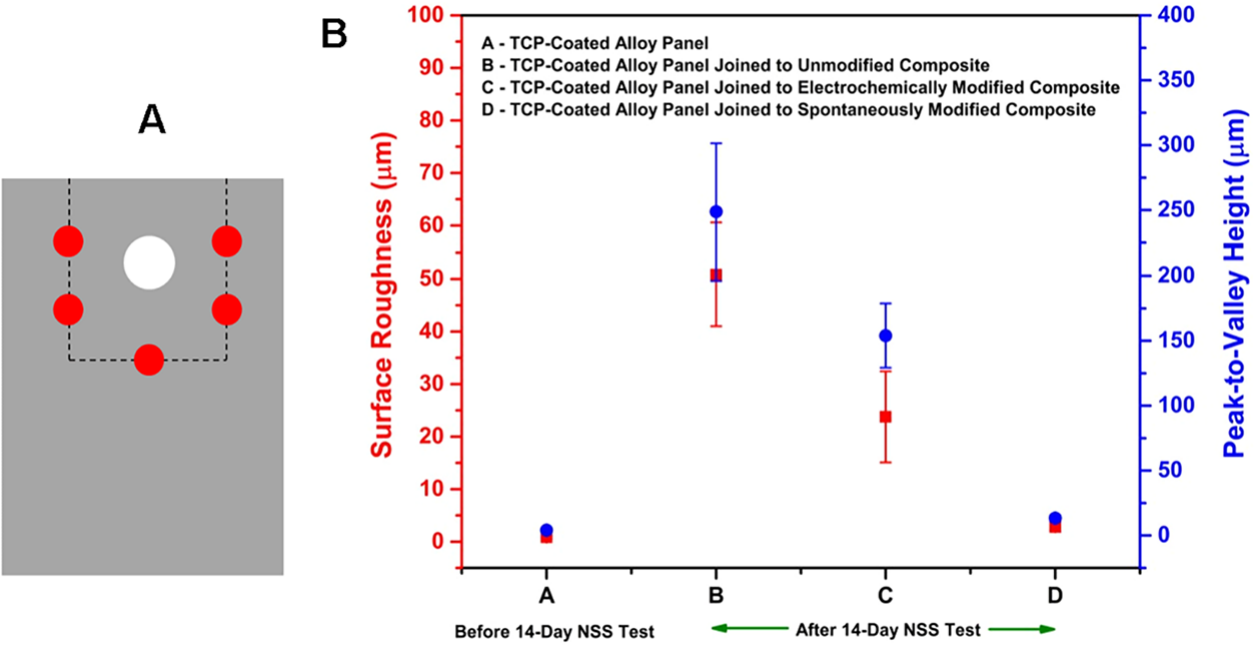
(A) Schematic diagram showing the areas where the surface topographical analysis was performed on a TCP-coated AA7075-T6 panel and (B) the average surface roughness (*S*_*q*_) and peak-to-valley height (*S*_*z*_) data for specimens before and after 14 days of NSS exposure. Data are presented for TCP-coated AA 7075-T6 panels joined with CFRP composites unmodified, electrochemically modified, and spontaneously modified (24-h immersion) with NAB. Data are presented as mean ± std. dev for *n* = 15, measurements per panel. Roughness data were recorded at 5 locations on each alloy panel with 3 panels of each type being used to generate the data.

The surface roughness measured for TCP-coated alloy panels prior to NSS exposure was 0.9 ± 0.3 μm. The surface roughness of the TCP-coated panels joined to an unmodified CFRP composite, and to a composite modified electrochemically and spontaneously with NAB, after 14 days of salt spray exposure were 51 ± 10, 24 ± 9, and 3 ± 1 μm, respectively. The largest surface roughness is seen for the TCP-coated panels joined to an unmodified CFRP composite due to the extensive galvanic corrosion damage that occurs. The surface roughness is 60× larger than the value for the unexposed control panel. The TCP-coated alloy panels joined with an electrochemically modified composite had a surface roughness increase of 28× with respect to the control panel and a surface roughness increase of only 2× was observed for TCP-coated alloy panels joined with a spontaneously modified composite. The peak-to-valley height data recorded for the unexposed alloy panel was 4.1 ± 0.7 μm. After 14 days of NSS exposure, TCP-coated panels joined to unmodified, electrochemically modified, and spontaneously modified CFRP composites had nominal peak-to-valley heights of 249 ± 53, 154 ± 25, and 13 ± 2 μm, respectively. These values reflect the galvanic corrosion that occurs leading to trench formation via metal dissolution, particularly on the specimens joined with unmodified and electrochemically modified composites. The peak-to-valley height is approximately 61× higher for the TCP-coated alloy panel joined to an unmodified composite, 38× higher for the TCP-coated alloy panel joined to an electrochemically modified composite, and merely 3× higher for the TCP-coated alloy panel joined to the spontaneously modified composite, as compared to the unexposed control panel.

Figure 12 presents weight loss and corrosion intensity (CI) data for TCP-coated AA7075-T6 panels joined with unmodified, electrochemically modified, and spontaneously modified CFRP composites after the 14-day NSS test. Recall that only one surface of the alloy panel was exposed to the salt spray. The TCP-coated panels joined with an unmodified composite exhibited the largest weight loss per unit area of ∼1.8 mg cm^–2^. The weight loss due to corrosion damage was ∼0.6 mg cm^–2^ or 67% lower for the TCP-coated panels coupled to an electrochemically modified composite. The smallest weight loss was observed for the TCP-coated panels joined to a composite surface treated with a spontaneously grafted NAB adlayer. This nominal weight loss was ∼0.1 mg cm^–2^, a 94× reduction as compared to a TCP-coated panel joined with an unmodified composite. The corrosion intensity, defined as the weight loss of the aluminum alloy per m^2^ per year, followed the same trend as the weight loss data and decreased in the following order: TCP-coated alloys coupled to an unmodified CFRP composite > TCP-coated alloys coupled to an electrochemically modified CFRP composite > TCP-coated alloys coupled to a spontaneously modified CFRP composite. Collectively, the NAB adlayer spontaneously formed on the composite edge during a 24-h immersion provides superior inhibition of the oxygen reduction reaction kinetics and therefore significantly attenuates galvanic corrosion on the joined alloy panel.

**Figure 12:**
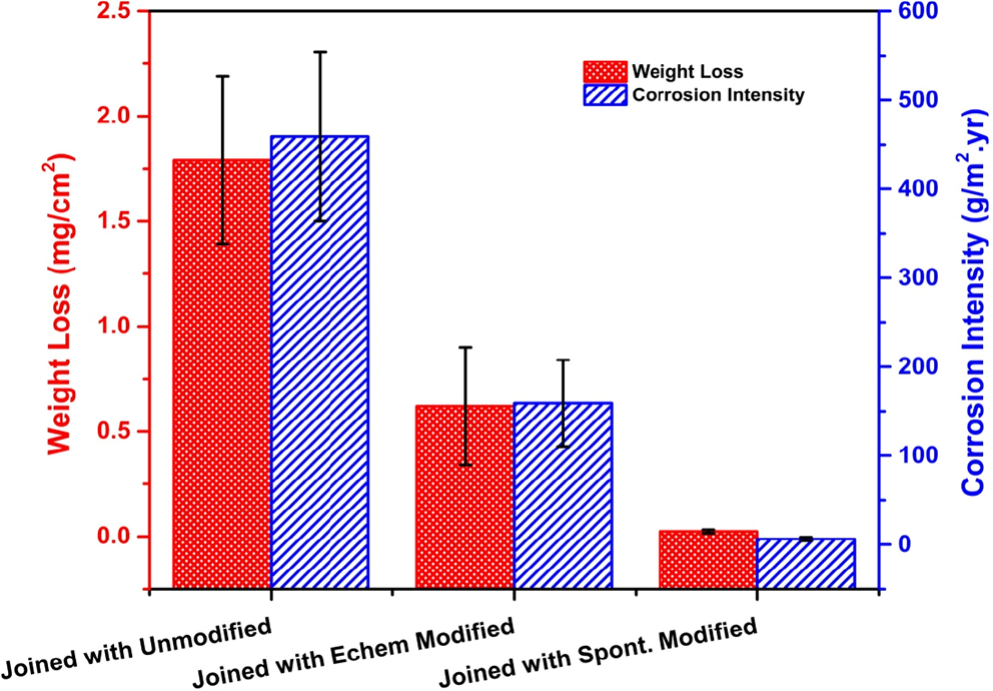
Weight loss and corrosion intensity data for TCP-coated AA7075-T6 panels joined with unmodified, electrochemically modified (NAB) and spontaneously modified (NAB) CFRP composites after 14 days of NSS exposure. Data are presented as mean ± std. dev. for *n* = 3 specimens of each type.

## Discussion

The treatment of exposed carbon fiber surfaces in a CFRP composite with a spontaneously formed organic adlayer of 4-nitroazobenzene (NAB) diazonium and its effect on mitigating galvanic corrosion damage on mechanically joined TCP-coated AA7075-T6 panels were studied. The surface treatment involved immersing freshly abraded, polished, and cleaned composite specimen edges and fastener through-holes in a solution of naturally aerated 5 mmol L^–1^ NAB dissolved in purified acetonitrile under open circuit conditions. The immersion time was 24 h. Specifically, the effectiveness of the surface treatment was assessed during a 14-day cyclic accelerated degradation test (TLM). This work builds upon our prior published work in two ways:^34,35^ (i) it clearly shows the benefit of the composite surface treatment for reducing galvanic corrosion on a different aluminum alloy, AA7075-T6, and (ii) it demonstrates the performance during a cyclic accelerated degradation test with conditions that are more representative of a real service environment.

The cyclic TLM conditions include being covered with a mist layer containing salt in equilibrium with the air, droplets of spray, condensation and precipitation, and heating and cooling.^6,43^ The atmosphere will control the initiation and growth of various forms of corrosion by changing the local chemical environment and the solution resistance between the anodic and cathodic regions on the alloy, or the alloy (anode) and exposed edges (cathode) of a joined CFRP composite. The TLM test uses a less aggressive salt solution (3.5 vs 5 wt. % NaCl as in NSS) but a higher temperature,incorporates some thermal cycling (23 h at 55 °C, cool down and 1 h at room temperature) and involves some solution/droplet evaporation and multiple rewettings during the test.^43^

The traditional method for forming diazonium adlayers on carbon and metal electrodes is through an electrochemically assisted process.^23–29,34,37–41^ The adlayers can also be formed spontaneously via electron transfer from the surface to the diazonium molecule dissolved in either aqueous or organic solution.^29–35,42^ Aryl diazonium adlayers have been formed spontaneously on a variety of carbon and metal electrodes.^23–29,34,35,37–41^ The spontaneous or “open circuit” formation is advantageous for practical application because it is adaptable in the carefully controlled aerospace workflow of joining a CFRP composite to an aluminum alloy. Variables including the reaction or immersion time, reduction potential of the aryldiazonium salt, its concentration, and the choice of solvent will likely affect the final surface coverage and morphology of the organic adlayer.^29–33,36–40^ These variables remain to be investigated.

The spontaneous formation of an aryl diazonium adlayer on the exposed carbon surfaces is a process in which aromatic diazonium cations (ArN_2_^+^) in solution are grafted onto the electrode surface without the need for an external electrical driving force. This spontaneous electron exchange involves the reduction of a diazonium molecule near the surface, leading to homolytic bond cleavage and the formation of an aryl radical (Ar^•^) upon the release of nitrogen gas (N_2_). The aryl radical then reacts with the carbon surface forming a covalent bond. The simplest explanation for this is that an electron transfers from the electrode to the diazonium molecule in solution because the lowest unoccupied molecular orbital (LUMO) is at a lower energy level (Fermi level) than the electron that resides in an occupied electronic state on the electrode. The transfer of the electron between the two would be thermodynamically favorable in this case and the difference in energy would represent the driving force for the electron exchange. This spontaneous process produces a stable adlayer of aromatic molecules on the electrode surface that is resistant to extended ultrasonic treatment and remains on the carbon fiber surface after the 14-day NSS accelerated degradation test.^34,35^

Importantly, the results presented herein demonstrate that a significant reduction in the galvanic corrosion rate of a TCP-coated aluminum alloy can be achieved when the exposed carbon fibers of a CFRP composite (i.e., the cut edges and the through-hole wall) are surface treated with a spontaneously formed NAB adlayer for 24 h. This was assessed using digital optical micrographs, surface topographic analysis, weight loss metrics, and corrosion intensity data. Minimal galvanic corrosion damage was observed on a TCP-coated alloy when joined with a composite specimen spontaneously modified with NAB after 14 days of the cyclic accelerated degradation test (TLM) or the legacy neutral salt spray exposure (NSS). The covalently bonded and stable adlayer is likely only 1 or 2 molecular layers thick (∼5 nm) but provides cathodic protection by blocking sites on the carbon fiber surface that are involved in the reduction of dissolved oxygen. The adlayer also serves as a barrier to direct contact of the underlying carbon with the environment. The oxygen reduction is a surface sensitive redox reaction on carbon electrodes.^59–62^ In the absence of the adlayer, O_2_ molecules chemisorb to active sites on the carbon fiber surface. This interaction serves to weaken the O=O bond, thus lowering the activation energy for the electrochemical reaction and increasing the heterogeneous electron-transfer rate constant. The NAB adlayer effectively blocks this surface interaction, which leads to a decrease in the heterogeneous electron-transfer rate. The blocking effect of diazonium adlayers toward surface-sensitive electrochemical reactions on carbon electrodes is well documented.^24,26,27,29–31,33–37,39,40^

Cyclic voltammetric data confirmed the presence of covalently bonded NAB admolecules on the carbon fiber surfaces. The nominal electrochemically active surface coverage for an adlayer formed during a 24 h immersion was 4.3 ± 0.4 nmol cm^–2^, which is consistent with the surface coverage of 3.1 ± 1.1 nmol cm^–2^ reported previously for a CFRP edge^34,35^ but higher than the theoretical monolayer coverage of 1.25 nmol cm^–2^.^48,49^ These values are normalized to the geometric area of the exposed edge and not the real surface area. The larger than monolayer surface coverage may indicate multilayer diazonium formation at least in some regions.^24,27,33,44,45^ The static contact angle measurements for water revealed that the spontaneously formed NAB adlayer decreases the wettability by increasing the hydrophobicity of the carbon fiber surface. The level of hydrophobicity imparted by the adlayer can be tailored as needed by changing the substituent groups of the aryl diazonium salt used.

The key finding from this work is the significantly reduced galvanic corrosion damage on a TCP-coated AA7075-T6 alloy when joined to a CFRP composite surface treated with a spontaneously formed NAB adlayer. The corrosion damage near the composite edge and the metal dissolution in and around the fastener through-hole were significantly less than the damage observed on alloy panels joined with an unmodified composite after the 14-day cyclic accelerated degradation test. In fact, surface topographical analysis (Figure 7) revealed little metal dissolution near a composite edge and underneath the composite on a TCP-coated alloy joined with a composite spontaneously modified with a NAB adlayer. The fastener hole damage was significantly reduced on the alloy joined with a composite spontaneously modified with NAB (24 h) (Figure 8). Consistent with the topographical analysis are the weight loss and corrosion intensity metrics which were both 84% lower than the values for the alloy joined with an unmodified composite (Figure 9).

Comparison studies of lap joint specimens exposed to a 14-day legacy NSS accelerated degradation test revealed similar findings about the benefits of the NAB surface treatment and are consistent with our prior published work on AA2024-T3.^34,35^ Digital optical micrographs reveal significantly less corrosion damage on a TCP-coated AA7075-T6 alloy in the forms of trenching near the exposed composite edges and crevice corrosion underneath the composite (Figure 10). Additionally, there is much less galvanic corrosion damage near and along the walls of the fastener through hole. The topographical surface roughness (*S*_*q*_) and peak-to-valley height (*S*_*z*_) parameters (Figure 11) were only 2–3× higher after the 14-day NSS test, as compared to values for an alloy joined to an unmodified CFRP composite which were 60× higher. The spontaneously formed NAB adlayer provides better blocking of the O_2_ reduction reaction, than does an electrochemically formed adlayer based on the topographical analysis (Figure 11) and the weight loss and corrosion intensity data (Figure 12). The spontaneously formed adlayer is less defective and more continuous over the carbon fiber surface than is the electrochemically formed adlayer. The weight loss (mg cm^–2^) and corrosion intensity values (g m^–2^ y^–1^) (Figure 12) were 94× lower than the values for an alloy joined to an unmodified CFRP composite.

## Conclusions

The NAB adlayer spontaneously grafted on the exposed carbon fiber surfaces of a CFRP composite by a 24-h immersion significantly attenuated galvanic corrosion on a joined TCP-coated AA7075-T6 alloy specimen during a 14-day (i) cyclic accelerated degradation test, so-called thin layer mist (TLM) and (ii) legacy neutral salt (NSS) exposure. Superior galvanic corrosion resistance is imparted by the NAB adlayer, which slows the rate of dissolved oxygen reduction on the nearby exposed CFRP composite fibers (i.e., cathodic protection). The key findings from the work can be summarized as follows:

1. NAB adlayers can be formed (24-h immersion) spontaneously on exposed carbon fibers of a CFRP composite without electrochemical assistance.
2. The spontaneously formed NAB adlayer increases the hydrophobicity of the CFRP composite by functioning as a moisture repellent barrier.
3. The electrochemically active surface coverage was 4.3 ± 0.4 nmol cm^–2^ for the CFRP composite spontaneously modified with NAB, which is higher than the theoretical monolayer coverage of 1.25 nmol cm^–2^. The higher apparent surface coverage is attributed to multilayer coverage.
4. There was far less galvanic corrosion damage near and along the walls of the fastener through holes on TCP-coated AA7075-T6 alloys joined with a spontaneously modified CFRP composite after the 14-day TLM and NSS accelerated degradation tests.
5. Galvanic corrosion in the form of trenching near the composite edges and crevice corrosion underneath the composite were significantly less after both 14-day accelerated degradation tests on a TCP-coated AA7075-T6 alloy when the composite was spontaneously modified with NAB.
6. After the 14-day TLM test, the weight loss and corrosion intensity metrics were both 84% lower for the alloy joined with a spontaneously modified composite as compared to the values for the alloy joined with an unmodified composite.
7. Topographical surface roughness (*S*_*q*_) and peak-to-valley height (*S*_*z*_) parameters were only 2–3× higher on TCP-coated alloys joined with a spontaneously modified composite after the 14-day NSS exposure, as compared to values for an alloy joined with an unmodified composite, which were 60× higher.
8. Weight loss (mg cm^–2^) and corrosion intensity (g m^–2^ y^–1^) parameters were 94% lower for TCP-coated AA7075-T6 alloys joined with a spontaneously modified CFRP composite than values for an alloy joined with an unmodified composite after the 14-day NSS test.

The spontaneously formed NAB adlayer provides better cathodic protection than does the electrochemically formed adlayer, ostensibly due to more complete coverage with fewer defects (i.e., better blocking layer).

## Data Availability

The paper includes much of the raw data generated in the project. Any other supporting data will be made available by the corresponding author upon reasonable request.
